# Psychological Distress Among Ethnically Diverse Participants From Eastern and Southern Africa

**DOI:** 10.1001/jamanetworkopen.2024.38304

**Published:** 2024-10-09

**Authors:** Kester B. B. Tindi, Allan Kalungi, Eugene Kinyanda, Bizu Gelaye, Alicia R. Martin, Ronald Galiwango, Wilber Ssembajjwe, Fred Kirumira, Adele Pretorius, Anne Stevenson, Charles R. J. C. Newton, Dan J. Stein, Elizabeth G. Atkinson, Emanuel K. Mwesiga, Joseph Kyebuzibwa, Lori B. Chibnik, Lukoye Atwoli, Mark Baker, Melkam Alemayehu, Rehema M. Mwende, Rocky E. Stroud, Solomon Teferra, Stella Gichuru, Symon M. Kariuki, Zukiswa Zingela, Moffat Nyirenda, Segun Fatumo, Dickens H. Akena

**Affiliations:** 1 Department of Immunology and Molecular Biology, College of Health Sciences, Makerere University, Kampala, Uganda; 2 School of Psychology and Vision Sciences, University of Leicester, Leicester, United Kingdom; 3 The African Computational Genomics Group, Medical Research Council/Uganda Virus Research Institute and London School of Hygiene and Tropical Medicine Uganda Research Unit, Entebbe, Uganda; 4 The African Center of Excellence in Bioinformatics and Data Intensive Sciences, Kampala, Uganda; 5 The Infectious Diseases Institute, Makerere University, Kampala, Uganda; 6 Department of Psychiatry, School of Medicine, College of Health Sciences, Makerere University, Kampala, Uganda, Entebbe, Uganda; 7 Medical Research Council/Uganda Virus Research Institute and London School of Hygiene and Tropical Medicine Uganda Research Unit; 8 Mental Health Section, Medical Research Council/Uganda Virus Research Institute and London School of Hygiene and Tropical Medicine Uganda Research Unit, Entebbe, Uganda; 9 Stanley Center for Psychiatric Research, Broad Institute of Harvard and MIT, Cambridge, Massachusetts; 10 Department of Epidemiology, Harvard T.H. Chan School of Public Health, Boston, Massachusetts; 11 The Chester M. Pierce MD Division of Global Psychiatry, Department of Psychiatry, Harvard Medical School and Massachusetts General Hospital, Boston; 12 Analytic and Translational Genetics Unit, Massachusetts General Hospital, Boston; 13 Program in Medical and Population Genetics, Broad Institute of Harvard and MIT, Cambridge, Massachusetts; 14 Data Section, Medical Research Council/Uganda Virus Research Institute and London School of Hygiene and Tropical Medicine Uganda Research Unit, Entebbe, Uganda; 15 College of Humanities and Social Sciences, Makerere University, Kampala, Uganda; 16 Department of Molecular and Human Genetics, Baylor College of Medicine, Houston, Texas; 17 Department of Mental Health, School of Medicine, Moi University College of Health Sciences, Eldoret, Kenya; 18 Brain and Mind Institute, The Aga Khan University, Nairobi, Kenya; 19 Department of Medicine, Medical College East Africa, The Aga Khan University, Nairobi, Kenya; 20 Department of Neurology, Massachusetts General Hospital, Harvard Medical School, Boston, Massachusetts; 21 Department of Psychiatry, Faculty of Medicine and Health Sciences, Stellenbosch University, Cape Town, South Africa; 22 Neurosciences Unit, Clinical Department, KEMRI-Wellcome Trust Research Programme-Coast, Kilifi, Kenya; 23 Department of Psychiatry, University of Oxford, Oxford, United Kingdom; 24 South African Medical Research Council (SAMRC) Unit on Risk and Resilience in Mental Disorders, Department of Psychiatry and Neuroscience Institute, University of Cape Town, Cape Town, South Africa; 25 Department of Psychiatry, School of Medicine, College of Health Sciences, Addis Ababa University, Addis Ababa, Ethiopia; 26 Executive Dean’s Office, Faculty of Health Sciences, Nelson Mandela University, Port Elizabeth, South Africa; 27 Department of Non-communicable Disease Epidemiology (NCDE), London School of Hygiene and Tropical Medicine, London, United Kingdom; 28 Department of Mental Health, Moi Teaching and Referral Hospital, Eldoret, Kenya; 29 Precision Healthcare University Research Institute, Queen Mary University of London, United Kingdom

## Abstract

**Importance:**

Psychological distress is characterized by anxiety and depressive symptoms. Although prior research has investigated the occurrence and factors associated with psychological distress in low- and middle-income countries, including those in Africa, these studies’ findings are not very generalizable and have focused on different kinds of population groups.

**Objective:**

To investigate the prevalence and characteristics (sociodemographic, psychosocial, and clinical) associated with psychological distress among African participants.

**Design, setting, and participants:**

This case-control study analyzed data of participants in the Neuropsychiatric Genetics in African Populations-Psychosis (NeuroGAP-Psychosis) study, which recruited from general outpatient clinics in Eastern (Uganda, Kenya, and Ethiopia) and Southern (South Africa) Africa. Individuals who participated in the control group of NeuroGAP-Psychosis from 2018 to 2023 were analyzed as part of this study. Data were analyzed from May 2023 to January 2024.

**Main outcomes and measures:**

The prevalence of psychological distress was determined using the Kessler Psychological Distress Scale (K10), which measures distress on a scale of 10 to 50, with higher scores indicating more distress. Participants from the NeuroGAP-Psychosis study were categorized into cases as mild (score of 20-24), moderate (score of 25-29), and severe (score of 30-50), and participants with scores less than 20 were considered controls. Factors that were associated with psychological distress were examined using binomial logistic regression.

**Results:**

From the data on 21 308 participants, the mean (SD) age was 36.5 (11.8) years, and 12 096 participants (56.8%) were male. The majority of the participants were married or cohabiting (10 279 participants [48.2%]), most had attained secondary education as their highest form of learning (9133 participants [42.9%]), and most lived with their families (17 231 participants [80.9%]). The prevalence of mild, moderate, and severe psychological distress was 4.2% (869 participants), 1.5% (308 participants), and 0.8% (170 participants), respectively. There were 19 961 participants (93.7%) who served as controls. Binomial logistic regression analyses indicated that the independent associations of psychological distress were experience of traumatic events, substance use (alcohol, tobacco, or cannabis), the physical comorbidity of arthritis, chronic neck or back pain, and frequent or severe headaches.

**Conclusions and relevance:**

In this case-control study among ethnically diverse African participants, psychological distress was associated with traumatic stress, substance use, and physical symptoms. These findings were observed to be consistent with previous research that emphasizes the importance of traumatic events as a factor associated with risk for psychopathology and notes the frequent co-occurrence of conditions such as physical symptoms, depression, and anxiety.

## Key Points

**Question:**

What are the prevalence and associated factors of psychological distress among African populations from Eastern and Southern Africa, recruited from general outpatient clinics?

**Findings:**

This case-control study involving 21 308 participants, found that 4.2% of the participants experienced mild psychological distress, 1.5% experienced moderate distress, and 0.8% experienced severe distress. Significant factors associated with psychological distress included exposure to traumatic events, substance use, and physical comorbidities.

**Meaning:**

These findings support existing evidence of the significance of traumatic events as a factor associated with risk for psychopathology, along with frequent co-occurrence of conditions such as physical symptoms, depression, and anxiety.

## Teaser

This case-control study evaluates the prevalence of and factors associated with psychological distress among an ethnically diverse population of individuals from Eastern and Southern Africa.

## Introduction

*Psychological distress* is a term used to describe emotional suffering. It encompasses symptoms of anxiety and depression, is highly prevalent, and often presents with physical symptoms such as insomnia.^1,2,3^ Depression and anxiety symptoms are often comorbid, and depending on the severity, can lead to serious impairment.^4^ High scores on measures of psychological distress, such as the Kessler psychological distress scale (K-10), are associated with the presence of mental disorders.^1,5,6^

Studies have indicated psychological distress to be commonly occurring in Western countries; for example, prevalence of self-reported psychological distress has been observed to range from 5% to 20% among different European populations.^7^ A study among adults in the US observed an increase in psychological distress prevalence from 16.1% (1999-2000) to 22.6% (2017-2018) with a focus on working age.^8^ A 10.4% prevalence of psychological distress was observed among outpatients in a general hospital in China, of which a greater proportion had higher psychological distress when comorbid with somatic distress.^9^

Psychological distress may be associated with multiple risk factors including sex, marital status, living areas, physical comorbidities, socioeconomic status, psychological factors, and so forth.^1,10,11,12,13,14^ However, the majority of these associations were observed in high income countries. Previous studies have been carried out on prevalence and correlates of psychological distress among low- and middle-income countries, including African countries, but findings have varied and were based on different kinds of populations.^2,11,15,16,17,18^ Therefore, this study aimed to investigate the prevalence and factors associated with risk of psychological distress among an ethnically diverse sample of participants recruited from general outpatient clinics from Eastern (Uganda, Kenya, and Ethiopia) and Southern (South Africa) Africa. Knowing the prevalence and correlates can enable the understanding of the underlying burden of psychological distress in these populations and better inform intervention strategies

## Methods

### Study Design

This case-control study of psychological distress among ethnically diverse participants was carried out using data on the control participants of the NeuroGAP-Psychosis study (2018-2023).^19^ In brief, NeuroGAP-Psychosis is a neuropsychiatric genetics study that is investigating the genetic risk of psychosis (schizophrenia or bipolar disorder) among participants in Uganda, Kenya, Ethiopia, and South Africa.^19^ The NeuroGAP-Psychosis study’s control participants were recruited from general outpatient clinics and were assessed for psychological distress using the K10, which has a scale of 10 to 50, with higher scores indicating more psychological distress.^20^ The participants were also assessed for their capacity to consent for clinical research using the University of California, San Diego Brief Assessment of Capacity to Consent (UBACC), and only those who scored above a cutoff of 14.5 were retained.^21^ Sociodemographic information including ethnicity was obtained through questionnaire-based interviews from which participant responses were noted and coded by research assistants.^19^ Some clinical data (HIV and body mass index [BMI]) were obtained through direct measurement. Some assessments were carried out using different specialized tools, such as the life events checklist for *Diagnostic and Statistical Manual of Mental Disorders* (Fifth Edition) for assessing traumatic life events^22^; the composite international diagnostic interview screener for chronic conditions,^23^ which was used to assess arthritis, chronic neck, or back pain; and frequent or severe headaches. The alcohol, smoking and substance screening test, version 3.0 was used to screen for use of alcohol, tobacco, and cannabis^24^ in the study. The phenotypic assessment tools used in the NeuroGAP were chosen based on several criteria: domain being assessed, cross-cultural validity, use of nonproprietary measures, the investigator’s experience with the tool, and time required to administer it.^19,25^ The current study obtained ethical approval from the Department of Immunology and Molecular Biology and the School of Biomedical Sciences Higher Degrees research ethics committee at Makerere University. The study also received a waiver of consent because consent was provided in the parent study and only deidentified secondary data were used in the current study. Design and reporting of this current study adhered to the Strengthening the Reporting of Observational Studies in Epidemiology (STROBE) reporting guideline for case-control studies^26^

### Definition of Cases and Controls

All NeuroGAP-Psychosis study’s control participants with a K10 score of 19 or lower were categorized as controls, those with a K10 score of 20 to 24 were categorized as mild cases, those with a K10 score of 25 to 29 were categorized as moderate cases, and those with a K10 score of 30 to 50 were classified as severe cases. These cutoffs are the ones recommended by the scale developers.^20^

### Statistical Analysis

Binomial logistic regression was used to carry out both univariate and multivariable association analyses between the dependent variable (psychological distress) and independent factors measured using odds ratios (OR). The analyses were adjusted to consider potential confounding effects stemming from country of birth, sex, and age on psychological distress.^1,27,28^ The factors investigated in this study were grouped as sociodemographic, psychosocial, and clinical factors. The significantly associated factors based on a *P* value threshold of *P* ≤ .003 after correcting for multiple testing using Bonferroni correction on 15 independent tests (*P* < .05/15) from the univariate analyses were retained in the multivariable analysis to observe their independent effects on psychological distress. Additionally, we carried out proportional odds logistic regression to observe the associations between the covariates and psychological distress while considering the increasing severity of psychological distress diagnoses from mild to severe. Data were analyzed with R version 4.2.3 (R Project for Statistical Computing) from May 2023 to January 2024.

## Results

### Phenotype Analysis

These analyses were carried out on 21 308 control participants from the NeuroGAP. Participants recruited from Ethiopia made up the biggest proportion (6497 participants [30.5%]), followed by Uganda (5991 participants [28.2%]), South Africa (4888 participants [22.9%]), and Kenya (3932 participants [18.5%]) (eTable 1 in Supplement 1). Male participants (12 096 participants [56.8%]) made up a bigger portion of the study population than female participants in all countries except Uganda (3200 female participants [53.4%]) (Table 1; eTable 1 in Supplement 1). The mean (SD) age for all participants was 36.5 (11.8) years, and most individuals were in the 25 to 34 year age group (6638 participants [31.6%]) (Table 1; eTable 1 in Supplement 1). Most participants were either married or cohabiting with their partners (10 279 participants [48.2%]), had attained secondary education as their highest form of learning (9133 participants [42.9%]), and lived with their family (17 231 participants [80.9%]) (Table 1).

**Table 1.** Sociodemographic Factors Associated With Psychological Distress (PD)

Sociodemographic factor	Participants, No. (%)	aOR (95% CI)^a^		
		Mild PD^b^	Moderate PD^b^	Severe PD^b^
Sex				
Female	9212 (43.2)	1.48 (1.29-1.70)^c^	1.67 (1.33-2.10)^c^	1.90 (1.39-2.61)^c^
Male	12 096 (56.8)	1 [Reference]	1 [Reference]	1 [Reference]
Age, y^d^				
18-24	3578 (17.0)	1 [Reference]	1 [Reference]	1 [Reference]
25-34	6638 (31.6)	1.18 (0.95-1.48)	1.22 (0.83-1.82)	1.78 (1.01-3.37)
35-44	5932 (28.2)	1.17 (0.93-1.47)	1.36 (0.93-2.04)	1.77 (0.99-3.37)
≥45	4857 (23.1)	1.20 (0.95-1.51)	1.47 (1.00-2.01)	2.59 (1.48-4.85)^c^
Marital status^d^				
Married or cohabits with partner	10 279 (48.2)	1 [Reference]	1 [Reference]	1 [Reference]
Never been married	8276 (38.8)	0.95 (0.80-1.14)	1.17 (0.88-1.55)	1.15 (0.76-1.72)
Divorced or separated	1941 (9.1)	1.22 (0.96-1.53)	1.37 (0.94-1.97)	2.33 (1.50-3.57)^c^
Widowed	810 (3.8)	1.26 (0.89-1.75)	1.31 (0.73-2.20)	1.76 (0.88-3.27)
Living arrangement^d^				
Lives with family	17 231 (80.9)	1 [Reference]	1 [Reference]	1 [Reference]
Lives alone	3479 (16.3)	1.06 (0.89-1.27)	0.94 (0.69-1.26)	1.33 (0.92-2.21)
Lives with friend or other	595 (2.8)	1.16 (0.74-1.74)	1.46 (0.72-2.67)	1.19 (0.36-2.87)
Education^d^				
No education	519 (2.4)	1 [Reference]	1 [Reference]	1 [Reference]
Primary education	4807 (22.6)	0.67 (0.45-1.02)	0.66 (0.34-1.45)	1.23 (0.49-4.14)
Secondary education	9133 (42.9)	0.49 (0.33-0.76)^c^	0.47 (0.24-1.03)	0.86 (0.34-2.92)
College education	6844 (32.1)	0.40 (0.27-0.63)^c^	0.45 (0.22-0.99)	0.72 (0.27-2.49)

Abbreviation: aOR, adjusted odds ratio.

^a^ Analyses were adjusted for age, sex, and country of birth.

^b^ The prevalence of psychological distress was determined using the Kessler Psychological Distress Scale, which measures distress on a scale of 10 to 50, with higher scores indicating more distress.

^c^ Statistically significant associations from the analyses after correcting for multiple testing.

^d^ Due to missing data for age (303 participants), marital status (2 participants), living arrangement (3 participants), and education (5 participants), these factors do not add up to the total sample size.

Most participants in this study scored below 20 on the K10, and thus were categorized as controls (19 961 participants [93.7%]) (eFigure in Supplement 1). Mild psychological distress had the highest prevalence among the different phenotypes (869 cases [4.2%]), followed by moderate psychological distress (308 cases and [1.5%]) and severe psychological distress (170 cases and [0.8%]). The highest prevalence across all psychological distress categories was observed in South African participants, and the lowest prevalence was observed in Ethiopian participants (eTable 2 in Supplement 1).

### Sociodemographic Factors Associated With Psychological Distress

The sociodemographic factors investigated in this study included sex, age, living arrangement, and highest educational level attained (Table 1). The factors significantly associated with mild psychological distress included female sex (adjusted OR [aOR], 1.48; 95% CI, 1.29-1.70; adjusted for age and country of birth), college level (aOR, 0.40; 95% CI, 0.27-0.63), and secondary level (aOR, 0.49; 95% CI, 0.33-0.76) education. The factors that were associated with moderate psychological distress included female sex (aOR, 1.67; 95% CI, 1.33-2.10; adjusted for age and country of birth). Severe psychological distress was found to be significantly associated with female sex (aOR, 1.90; 95% CI, 1.31-2.61; adjusted for age and country of birth), age (≥45 years) (aOR, 2.59; 95% CI, 1.48-4.85; adjusted for sex and country), and being divorced (aOR, 2.33; 95% CI, 1.50-3.57), which showed a significant positive association compared with being married.

### Psychosocial Factors Associated With Psychological Distress

The psychosocial factors investigated in this study included types of traumatic events (witnessed or experienced) and substance abuse (alcohol, tobacco, and cannabis) (Table 2). The majority of participants had witnessed (13 404 participants [63.7%]) and experienced (11 786 participants [55.9%]) at least 1 traumatic event. The highest proportion of participants had never used alcohol (8017 participants [37.6%]), tobacco (15 176 participants [71.2%]), or cannabis (18 267 participants [85.7%]).

**Table 2.** Psychosocial Factors Associated With Psychological Distress (PD)

Psychosocial factor	Participants, No. (%)	aOR (95% CI)^a^		
		Mild PD^b^	Moderate PD^b^	Severe PD^b^
Traumatic life events				
Witnessed by participant^c^				
0 events	7648 (36.3)	1 [Reference]	1 [Reference]	1 [Reference]
≥1 event	13 404 (63.7)	2.61 (2.10-3.26)^d^	2.33 (1.58-3.50)^d^	1.35 (0.86-2.15)
Experienced by participant^c^				
0 events	9294 (44.1)	1 [Reference]	1 [Reference]	1 [Reference]
≥1 event	11 786 (55.9)	2.94 (2.42-3.59)^d^	3.42 (2.39-5.00)^d^	3.71 (2.31-6.26)^d^
Substance abuse				
Alcohol use				
Never used alcohol	8017 (37.6)	1 [Reference]	1 [Reference]	1 [Reference]
Daily or almost daily	699 (3.3)	2.17 (1.48-3.12)^d^	2.76 (1.53-4.70)^d^	3.41 (1.62-6.65)^d^
Weekly	3106 (14.6)	1.33 (1.08-1.68)	1.04 (0.71-1.53)	0.74 (0.40-1.31)
Monthly	1719 (8.1)	1.36 (1.03-1.78)	0.88 (0.52-1.41)	1.44 (0.78-2.58)
Once or twice in the past 3 mo	2714 (12.7)	1.21 (0.95-1.53)	1.16 (0.78-1.74)	1.39 (0.83-2.29)
None used	5053 (23.7)	1.45 (1.19-1.76)^d^	1.33 (0.96-1.83)	1.36 (0.89-2.08)
Tobacco use				
Never used alcohol	15 176 (71.2)	1 [Reference]	1 [Reference]	1 [Reference]
Daily or almost daily	2747 (12.9)	1.67 (1.35-2.05)^d^	1.74 (1.27-2.41)^d^	3.61 (2.30-5.69)^d^
Weekly	317 (1.5)	2.22 (1.41-3.36)^d^	1.54 (0.64-3.16)	0.65 (0.04-3.03)
Monthly	88 (0.4)	2.16 (0.89-4.48)	2.32 (0.55-6.48)	7.21 (1.70-20.71)^d^
Once or twice in the past 3 mo	277 (1.3)	1.53 (0.85-2.54)	1.30 (0.46-2.94)	3.08 (0.92-7.68)
None used	2703 (12.7)	1.37 (1.10-1.70)^d^	1.09 (0.73-1.58)	1.52 (0.87-2.52)
Cannabis use				
Never used alcohol	18 267 (85.7)	1 [Reference]	1 [Reference]	1 [Reference]
Daily or almost daily	486 (2.3)	1.73 (1.21-2.41)^d^	2.98 (1.85-4.66)^d^	4.87 (2.52-8.95^)^^d^
Weekly	218 (1.0)	1.44 (0.81-2.37)	2.67 (1.52-4.90)^d^	4.21 (1.58-9.40)^d^
Monthly	92 (0.4)	2.61 (1.31-4.78)	0.54 (0.30-2.47)	3.28 (0.53-11.10)
Once or twice in the past 3 mo	277 (1.3)	2.09 (1.33-3.15)^d^	1.66 (0.73-3.26)	4.14 (1.08-2.78)^d^
None used	1968 (9.2)	1.53 (1.24-1.88)^d^	1.27 (0.90-1.78)	1.76 (1.08-2.78)

Abbreviation: aOR, adjusted odds ratio.

^a^ Analyses were adjusted for age, sex, and country of birth.

^b^ The prevalence of psychological distress was determined using the Kessler Psychological Distress Scale, which measures distress on a scale of 10 to 50, with higher scores indicating more distress.

^c^ Due to missing data for traumatic life events witnessed (256 participants) and traumatic life events experienced (228 participants), these factors do not add up to the total sample size.

^d^ Statistically significant associations from the analyses after correcting for multiple testing.

The psychosocial factors significantly associated with mild psychological distress included witnessing at least 1 type of traumatic life event (aOR, 2.61; 95% CI, 2.10-3.26) and personally experiencing at least 1 type of traumatic life event (aOR, 2.94; 95% CI, 2.42-3.59). Daily alcohol use (aOR, 2.17; 95% CI, 1.48-3.12), and no alcohol used in the past 3 months (aOR, 1.45; 95% CI, 1.19-1.76) were significantly associated with mild psychological distress compared with having never used alcohol. Daily tobacco use (aOR, 1.67; 95% CI, 1.35-2.05) and weekly tobacco use (aOR, 2.22; 95% CI, 1.41-3.36) both showed statistically significant associations with mild psychological distress compared with having never used tobacco. Daily cannabis use (aOR, 1.73; 95% CI, 1.21-2.41), cannabis use once or twice in the past 3 months (aOR, 2.09; 95% CI, 1.33-3.15), and no cannabis used in the past 3 months (aOR, 1.53; 95% CI, 1.24-1.88) all showed statistically significant associations with mild psychological distress compared with having never used cannabis.

The adjusted psychosocial factors significantly associated with moderate psychological distress included witnessing at least 1 type of traumatic life event (aOR, 2.33; 95% CI, 1.58-3.50) and personally experiencing at least 1 type of traumatic life event (aOR, 3.42; 95% CI, 2.39-5.00). Daily alcohol use (aOR, 2.76; 95% CI, 1.53-4.70), daily tobacco use (aOR, 1.74; 95% CI, 1.27-2.41), and daily (aOR, 2.98; 95% CI, 1.85-4.66) and weekly (aOR, 2.67; 95% CI, 1.52-4.90) cannabis use all showed statistically significant higher odds of moderate psychological distress.

The adjusted psychosocial factors significantly associated with severe psychological distress included personally experiencing at least 1 type of traumatic life event (aOR, 3.71; 95% CI. 2.31-6.26) compared with not experiencing an event. Daily alcohol use (aOR, 3.41; 95% CI, 1.62-6.65), daily tobacco use (aOR, 3.61; 95% CI, 2.30-5.69) and monthly tobacco use (aOR, 7.21; 95% CI, 1.70-20.01) all showed significantly higher odds of severe psychological distress. Daily cannabis use (aOR, 4.87; 95% CI, 2.52-8.95), weekly cannabis use (aOR, 4.21; 95% CI, 1.58-9.40) and cannabis use once or twice in the past 3 months (aOR, 4.14; 95% CI, 1.08-2.78) all showed statistically significant higher odds of severe psychological distress compared with not using cannabis.

### Clinical Factors Associated With Psychological Distress

The clinical factors that were investigated in this study included the physical comorbidities of chronic back or neck pain, frequent or severe headaches, arthritis, and HIV. We also investigated BMI in these analyses (Table 3). The majority (12 283 participants [57.7%]) of study participants had a BMI between 18.5 and 24.9, 7218 (33.9%) were either overweight (25.0-29.9) or obese (≥30), and 1798 (8.4%) were underweight (<18.5). With regards to comorbidities, 2146 (10.3%) were HIV positive, 1589 (7.5%) had arthritis, 2915 (13.7%) had chronic back or neck pain, and 4187 (19.7%) had frequent or severe headaches.

**Table 3.** Clinical Factors Associated With Psychological Distress (PD)

Clinical factor	Participants, No. (%)	aOR (95% CI)^a^		
		Mild PD	Moderate PD	Severe PD
Body mass index^b^^,^^c^				
<18.5	1798 (8.4)	1.40 (1.08-1.79)	1.32 (0.83-2.01)	1.99 (1.14-3.30)
18.5-24.9	12 283 (57.7)	1 [Reference]	1 [Reference]	1 [Reference]
25.0-29.9	4652 (21.8)	0.92(0.76-1.10)	0.98 (0.72-1.31)	1.06 (0.70-1.60)
≥30	2566 (12.0)	0.98 (0.79-1.21)	0.98 (0.70-1.36)	1.12 (0.71-1.76)
HIV^c^				
HIV negative	5895 (28.3)	1 [Reference]	1 [Reference]	1 [Reference]
HIV positive	2146 (10.3)	0.98 (0.77-1.24)	1.00 (0.67-1.45)	1.10 (0.65-1.80)
HIV status unknown	12 801 (61.4)	0.76 (0.65-0.89)^d^	0.74 (0.58-0.98)	0.89 (0.63-1.28)
Arthritis				
No arthritis	19 582 (91.9)	1 [Reference]	1 [Reference]	1 [Reference]
Has arthritis	1589 (7.5)	2.15 (1.72-2.67)^d^	1.81 (1.25-2.57)^d^	3.35 (2.18-5.04)^d^
Arthritis status unknown	137 (0.6)	0.58 (0.25-1.56)	3.50 (1.61-6.70)^d^	5.71 (2.31-11.98)^d^
Chronic back or neck pain^c^				
No pain	18 359 (86.2)	1 [Reference]	1 [Reference]	1 [Reference]
Has pain	2915 (13.7)	2.47 (2.10-2.89)^d^	2.33 (1.80-2.99)^d^	3.38 (2.43-4.67)^d^
Pain status unknown	32 (0.2)	1.75 (0.28-6.01)	2.33 (0.13-11.41)	0.00 (0.00-1.9e +18)
Frequent or severe headaches^c^				
No headaches	17 088 (80.2)	1 [Reference]	1 [Reference]	1 [Reference]
Has headaches	4187 (19.7)	2.87 (2.48-3.31)^d^	2.99 (2.37-3.79)^d^	4.47 (3.26-6.17)^d^
Headache status unknown	32 (0.2)	2.72 (0.64-7.91)	2.41 (0.13-11.71)	0.00 (0.00-4.5e +17)

Abbreviation: aOR, adjusted odds ratio.

^a^ Analyses were adjusted for age, sex, and country of birth.

^b^ Calculated as weight in kilograms divided by height in meters squared.

^c^ Due to missing data for body mass index (9 participants), HIV (466 participants), chronic neck or back pain (2 participants), and frequent or severe headaches (1 participants), these factors do not add up to the total sample size.

^d^ Statistically significant associations from the analyses after correcting for multiple testing.

Having arthritis (aOR, 2.15; 95% CI, 1.72-2.76), chronic neck or back pain (aOR, 2.47; 95% CI, 2.10-2.89), and frequent or severe headaches (aOR, 2.87; 95% CI, 2.48-3.31) all showed significantly higher odds of mild psychological distress after adjusting for age, sex, and country of birth. Unknown HIV status (aOR, 0.76; 95% CI, 0.65-0.89) displayed significantly lower odds of mild psychological distress. Having chronic neck or back pain (aOR, 2.33; 95% CI, 1.80-2.99), having arthritis (aOR, 1.81; 95% CI, 1.25-2.57), unknown arthritis status (aOR, 3.50; 95% CI, 1.61-6.70), and frequent or severe headaches (aOR, 2.99; 95% CI, 2.37-3.79) all showed significantly higher odds of moderate psychological distress. Having arthritis (aOR, 3.35; 95% CI, 2.18-5.04), unknown arthritis status (OR, 3.38; 95% CI, 2.31-11.98), chronic neck or back pain (aOR, 5.71; 95% CI, 2.43-4.67), and frequent or severe headaches (aOR, 4.47; 95% CI, 3.26-6.17) all showed significantly higher odds of severe psychological distress after adjusting.

### Factors Significantly Associated With Psychological Distress in Multivariable and Proportional Odds Analyses

Witnessing or experiencing traumatic life events, daily cannabis use, having chronic neck or back pain, and having frequent or severe headaches were independently significantly associated with mild and moderate psychological distress (Table 4). Experiencing traumatic life events, tobacco and cannabis use, having arthritis, having chronic neck or back pain, and having frequent or severe headaches were associated with severe psychological distress (Table 4). Being female; witnessing or experiencing traumatic life events; alcohol, tobacco, and cannabis use; having arthritis; having chronic neck or back pain; and having frequent or severe headaches were associated with increasing severity of psychological distress (eTable 3 in Supplement 1).

**Table 4.** Factors Associated With Psychological Distress (PD) at Multivariable Analysis

Factor	OR (95% CI)		
	Mild PD	Moderate PD	Severe PD
Traumatic life events			
≥1 event witnessed by participant	1.89 (1.51-2.37)	1.70 (1.15-2.58)	0.84 (0.53-1.36)
≥1 event experienced by participant	2.18 (1.79-2.68)	2.62 (1.82-3.86)	2.67 (1.64-4.56)
Daily tobacco use	1.24 (0.98-1.58)	1.31 (0.90-1.90)	2.47 (1.46-4.17)
Daily cannabis use	1.27 (0.86-1.83)	2.32 (1.39-3.79)	2.79 (1.38-5.43)
Has arthritis	1.41 (1.11-1.77)	1.21 (0.82-1.75)	2.13 (1.35-3.31)
Has chronic back or neck pain	1.57 (1.32-1.86)	1.55 (1.17-2.04)	1.76 (1.22-2.53)
Has frequent or severe headaches	2.19 (1.87-2.56)	2.36 (1.83-3.03)	3.10 (2.19-4.38)

Abbreviation: OR, odds ratio.

## Discussion

Rates of mild, moderate, and severe psychological distress were observed at 4.2%, 1.5%, and 0.8%, respectively, and analyses showed traumatic events, the use of substances like tobacco or cannabis, as well as the presence of physical comorbidities such as arthritis, chronic neck or back pain, and frequent or severe headaches to be associated with psychological distress. The aforementioned factors were also observed to be significantly associated with increasing severity of psychological distress from mild to severe. Previous studies have found prevalence of psychological distress to range between 12.1% and 64.5% across African populations.^15,29,30,31,32,33,34^ However, there is a paucity of data on the prevalence of psychological distress within the Eastern and Southern African countries from whom participants were recruited as part of the parent study. Most of the studies within the medical context were carried out in specialized hospital wards (tuberculosis, cancer, and so forth) where people are more likely to be under distress due to the additional stressors that accompany chronic illnesses such as stigma, comorbidities, and taxing treatment strategies.^15,32,34^ It should be noted that cultural differences have been observed to have a substantial impact on the interpretation of the K10 items, which could explain the observed variations of prevalence by country in our study findings.^25^

Our findings of an association between psychological distress, traumatic events, substance use, and somatic symptoms are consistent with a range of previous work from high income countries. There is a smaller body of work on such associations in low-income countries, and our work contributes to an emphasis on the universal triad of depression, anxiety, and pain. There are, however, complex bidirectional causal relationships that contribute to these associations. For example, the significant association between witnessing or experiencing traumatic life events and psychological distress observed has been previously described in studies where the strength of association varies depending on the traumatic events experienced.^35,36^ Different traumatic event types have been observed to be associated with increased severity and higher risk of comorbidities in psychological distress. For example, traumatic exposures such as conflicts or wars (man-made disasters) have been observed to be associated with higher levels of psychological distress than natural disasters (eg, floods).^37,38^ Lifetime abuse (physical or sexual), especially in childhood, has been observed to be associated with severe psychological distress comorbid with pain and functional disability in a relationship mediated by traumatic stress.^39,40^ Tobacco smoking showed a statistically significant increased risk of severe psychological distress agreeing with previous studies that have shown tobacco smoking to be associated with more severe psychological distress, and individuals with mental health problems have been observed to smoke at a higher rate than the general population.^41,42^ More frequent cannabis use has been previously associated with more serious forms of psychological distress, which agrees with the findings of our study that showed association between daily cannabis use and moderate to severe psychological distress.^43^ Alcohol use was also significantly associated with increased severity of psychological distress in the proportional odds analysis, which is similar to previous studies that have observed greater psychological distress among individuals with higher alcohol consumption.^44,45^

Chronic neck or back pain showed increased risk of psychological distress and previous studies have found that there is a greater risk of psychological distress among people with neck or back pain.^46^ Frequent or severe headaches were significantly associated with an increased risk of psychological distress, which agrees with previous findings that showed headaches to be associated with psychological distress as well as anxiety and depressive symptoms.^47,48^ Arthritis showed a statistically significant increased risk of severe psychological distress, and some studies have found psychological distress to increase the risk of arthritis, while others have observed a positive association between the levels of stress, anxiety, and depression with arthritis.^49,50^ The findings of this study indicate that clinical correlates of psychological distress are similar in sub-Saharan Africa as have been described elsewhere, implying that established interventions that have been shown to be efficacious can be adapted and implemented successfully in African populations.

Future studies could look into the effect of specific traumatic events as different event types may have higher odds of psychological distress. Future studies could employ longitudinal study designs to establish the temporal order between risk factors and psychological distress, and thus ascertain the direction of effects. Both treatment and prognosis of psychological distress can be improved in these populations by ascertaining the direction of effects.

### Limitations

This study has limitations. We explored psychological distress within the control participants of the NeuroGAP-Psychosis study who were less likely to experience psychological distress, given the stringent exclusion criteria imposed by the parent study. The study comprised individuals recruited from general outpatient clinics in the 4 participant countries, encompassing outpatients, caretakers, students, hospital workers, and others. However, the inclusion of such a specific sample may limit the generalizability of the findings to the broader population. Cultural differences between participating countries were observed to impact interpretation of some items of the K10. Analyses involved 15 correlates of psychological distress and are thus subject to the pitfalls of multiple testing, such as false-positive results. Multiple testing was adjusted for using Bonferroni correction, but this method is highly conservative. Not every available risk factor from the NeuroGAP, including factors like chronic infections or specific traumatic events, were investigated due to the breadth of this study. Direction of effects require alternative study types such as longitudinal studies to be effectively determined.

## Conclusions

The associations observed with traumatic stress, substance use, and physical symptoms agree with previous study findings highlighting the key role of traumatic events in the development of psychological distress as well as its frequent comorbidities. The prevalence of psychological distress observed aligns with findings of some studies carried out in similar populations, but it should be noted that cultural differences can play a role in the interpretation of the K10 items, which possibly influences diagnosis and the prevalence observed. This study found that psychosocial factors, psychosomatic complaints, and physical comorbidities are associated with psychological distress among adult ethnically diverse participants from Eastern and Southern Africa.

## Notes

Supplement 1. **eFigure. **Distribution of Kessler Psychological Distress Scale Scores in the Study Participants **eTable 1. **Characterization of Study Population by Country **eTable 2. **Prevalence of Psychological Distress by Country **eTable 3. **Factors That Were Statistically Significant From the Proportional Odds Logistic Regression Analysis

Supplement 2.
Data Sharing Statement

## References

[1] Viertiö S, Kiviruusu O, Piirtola M, . Factors contributing to psychological distress in the working population, with a special reference to gender difference. BMC Public Health. 2021;21(1):611. doi:10.1186/s12889-021-10560-y 33781240 PMC8006634

[2] Belay AS, Guangul MM, Asmare WN, Mesafint G. Prevalence and associated factors of psychological distress among nurses in public hospitals, Southwest, Ethiopia: a cross-sectional study. Ethiop J Health Sci. 2021;31(6):1247-1256. 35392329 10.4314/ejhs.v31i6.21 PMC8968359

[3] Regier DA, Kuhl EA, Kupfer DJ. The DSM-5: Classification and criteria changes. World Psychiatry. 2013;12(2):92. doi:10.1002/wps.20050 23737408 PMC3683251

[4] Kalin NH. The critical relationship between anxiety and depression. Am J Psych. 2020;177(5):365-367. doi:10.1176/appi.ajp.2020.20030305 32354270

[5] Drapeau A, Marchand A, Beaulieu-Prevost D. Epidemiology of Psychological Distress. Mental Illnesses - Understanding, Prediction and Control. IntechOpen; 2012.

[6] Kessler RC, Barker PR, Colpe LJ, . Screening for serious mental illness in the general population. Arch Gen Psychiatry. 2003;60(2):184-189. doi:10.1001/archpsyc.60.2.184 12578436

[7] Health at a Glance: Europe 2018. OECD; 2018.

[8] Daly M. Prevalence of psychological distress among working-age adults in the United States, 1999-2018. Am J Public Health. 2022;112(7):1045-1049. doi:10.2105/AJPH.2022.306828 35617656 PMC9222463

[9] Xiong N, Wei J, Fritzsche K, . Psychological and somatic distress in Chinese outpatients at general hospitals: a cross-sectional study. Ann Gen Psychiatry. 2017;16(1):35. doi:10.1186/s12991-017-0158-y 29075308 PMC5644179

[10] Kim S, Jang HJ, Myung W, . Heritability estimates of individual psychological distress symptoms from genetic variation. J Affect Disord. 2019;252:413-420. doi:10.1016/j.jad.2019.04.011 31003110

[11] Ncitakalo N, Sigwadhi LN, Mabaso M, Joska J, Simbayi L. Exploring HIV status as a mediator in the relationship of psychological distress with socio-demographic and health related factors in South Africa: findings from the 2012 nationally representative population-based household survey. AIDS Res Ther. 2023;20(1):6. doi:10.1186/s12981-022-00498-5 36747255 PMC9901137

[12] Barry V, Stout ME, Lynch ME, . The effect of psychological distress on health outcomes: a systematic review and meta-analysis of prospective studies. J Health Psych. 2019;25(2):227-39. doi:10.1177/1359105319842931 30973027

[13] Knifton L, Inglis G. Poverty and mental health: policy, practice and research implications. BJPsych Bull. 2020;44(5):193. doi:10.1192/bjb.2020.78 32744210 PMC7525587

[14] Li Z, Zhang L. Poverty and health-related quality of life: a cross-sectional study in rural China. Health Qual Life Outcomes. 2020;18(1):153. doi:10.1186/s12955-020-01409-w 32456683 PMC7249398

[15] Naisanga M. SekaggyaWiltshire C, Muhwezi WW, Musaazi J, Akena D. Prevalence and factors associated with psychological distress among patients on warfarin at the Uganda Heart Institute, Mulago Hospital. BMC Psychiatry. 2022;22(1):1-9. doi:10.1186/s12888-022-03998-w 35596217 PMC9123720

[16] Nantaayi B, Ndawula RK, Musoke P, . Psychological distress and access to mental health services among undergraduate students during the COVID-19 lockdown in Uganda. Front Psychiatry. 2022;13:792217. doi:10.3389/fpsyt.2022.792217 35722591 PMC9201074

[17] MacGinty RP, Kariuki SM, Barnett W, . Associations of antenatal maternal psychological distress with infant birth and development outcomes: results from a South African birth cohort. Compr Psychiatry. 2020;96:152128. doi:10.1016/j.comppsych.2019.152128 31715335 PMC6945113

[18] Theron G, Peter J, Zijenah L, . Psychological distress and its relationship with non-adherence to TB treatment: a multicentre study. BMC Infect Dis. 2015;15(1):253. doi:10.1186/s12879-015-0964-2 26126655 PMC4487582

[19] Stevenson A, Akena D, Stroud RE, . Neuropsychiatric GENETICS of African Populations-Psychosis (NeuroGAP-Psychosis): a case-control study protocol and GWAS in Ethiopia, Kenya, South Africa and Uganda. BMJ Open. 2019;9(2):e025469. doi:10.1136/bmjopen-2018-025469 30782936 PMC6377543

[20] Kessler RC, Barker PR, Colpe LJ, Epstein JF, Gfroerer JC, Hiripi E. Screening for serious mental illness in the general population. Arch Gen Psychiatry. 2003;60(2):184-189. doi:10.1001/archpsyc.60.2.184 12578436

[21] Jeste DV, Palmer BW, Appelbaum PS, . A new brief instrument for assessing decisional capacity for clinical research. Arch Gen Psychiatry. 2007;64(8):966-974. doi:10.1001/archpsyc.64.8.966 17679641

[22] Gray MJ, Litz BT, Hsu JL, Lombardo TW. Psychometric properties of the life events checklist. Assessment. 2004;11(4):330-341. doi:10.1177/1073191104269954 15486169

[23] Kessler RC, Ustün TB. The World Mental Health (WMH) survey initiative version of the World Health Organization (WHO) Composite International Diagnostic Interview (CIDI). Int J Methods Psychiatr Res. 2004;13(2):93-121. doi:10.1002/mpr.168 15297906 PMC6878592

[24] Ali R, Awwad E, Babor TF, ; WHO ASSIST Working Group. The Alcohol, Smoking and Substance Involvement Screening Test (ASSIST): development, reliability and feasibility. Addiction. 2002;97(9):1183-1194. doi:10.1046/j.1360-0443.2002.00185.x 12199834

[25] Ametaj AA, Denckla CA, Stevenson A, . Cross-cultural equivalence of the Kessler Psychological Distress Scale (K10) across four African countries in a multi-national study of adults. SSM Ment Health. 2024;5:100300. doi:10.1016/j.ssmmh.2024.100300 38706931 PMC11064105

[26] von Elm E, Altman DG, Egger M, Pocock SJ, Gøtzsche PC, Vandenbroucke JP; STROBE Initiative. The Strengthening the Reporting of Observational Studies in Epidemiology (STROBE) statement: guidelines for reporting observational studies. Ann Intern Med. 2007;147(8):573-577. doi:10.7326/0003-4819-147-8-200710160-00010 17938396

[27] Atwoli L, Muhia J, Wanja Gitau C. From diversity to individualized care: Africa’s contribution to psychiatry. World Psychiatry. 2022;21(3):424-426. doi:10.1002/wps.21007 36073710 PMC9453884

[28] Best R, Strough J, Bruine de Bruin W. Age differences in psychological distress during the COVID-19 pandemic: March 2020 - June 2021. Front Psychol. 2023;14:1101353. doi:10.3389/fpsyg.2023.1101353 36814666 PMC9939750

[29] Anyanwu MU. Psychological distress in adolescents: prevalence and its relation to high-risk behaviors among secondary school students in Mbarara Municipality, Uganda. BMC Psychol. 2023;11(1):5. doi:10.1186/s40359-023-01039-z 36624544 PMC9830719

[30] Clarke-Deelder E, Rokicki S, McGovern ME, . Levels of depression, anxiety, and psychological distress among Ugandan adults during the first wave of the COVID-19 pandemic: cross-sectional evidence from a mobile phone-based population survey. Glob Ment Health (Camb). 2022;9:274-284. doi:10.1017/gmh.2022.28 36618739 PMC9807010

[31] Gebremedhin HT, Bifftu BB, Lebessa MT, Weldeyes AZ, Gebru TT, Petrucka P. Prevalence and associated factors of psychological distress among secondary school students in Mekelle City, Tigray Region, Ethiopia: cross-sectional study. Psychol Res Behav Manag. 2020;13:473-480. doi:10.2147/PRBM.S252779 32547269 PMC7250291

[32] Negussie F, Giru BW, Yusuf NT, Gela D. Psychological distress and associated factors among cancer patients in public hospitals, Addis Ababa, Ethiopia: a cross-sectional study. BMC Psychol. 2023;11(1):41. doi:10.1186/s40359-023-01079-5 36765415 PMC9921361

[33] Mthembu JC, Mabaso MLH, Khan G, Simbayi LC. Prevalence of psychological distress and its association with socio-demographic and HIV-risk factors in South Africa: findings of the 2012 HIV prevalence, incidence and behaviour survey. SSM Popul Health. 2017;3:658-662. doi:10.1016/j.ssmph.2017.07.009 29349254 PMC5769074

[34] Ayana TM, Roba KT, Mabalhin MO. Prevalence of psychological distress and associated factors among adult tuberculosis patients attending public health institutions in Dire Dawa and Harar cities, Eastern Ethiopia. BMC Public Health. 2019;19(1):1392. doi:10.1186/s12889-019-7684-2 31660912 PMC6819569

[35] Marum G, Clench-Aas J, Nes RB, Raanaas RK. The relationship between negative life events, psychological distress and life satisfaction: a population-based study. Qual Life Res. 2014;23(2):601-611. doi:10.1007/s11136-013-0512-8 24026629

[36] Nyarko F, Peltonen K, Kangaslampi S, Punamäki-Gitai RL. How stressful life events and violence are related to mental health: the protective role of social relations in African context. Heliyon. 2020;6(8):e04629. doi:10.1016/j.heliyon.2020.e04629 32802978 PMC7419586

[37] Makwana N. Disaster and its impact on mental health: a narrative review. J Family Med Prim Care. 2019;8(10):3090-3095. doi:10.4103/jfmpc.jfmpc_893_19 31742125 PMC6857396

[38] Beaglehole B, Mulder RT, Frampton CM, Boden JM, Newton-Howes G, Bell CJ. Psychological distress and psychiatric disorder after natural disasters: systematic review and meta-analysis. Br J Psychiatry. 2018;213(6):716-722. doi:10.1192/bjp.2018.210 30301477

[39] Sachs-Ericsson N, Cromer K, Hernandez A, Kendall-Tackett K. A review of childhood abuse, health, and pain-related problems: the role of psychiatric disorders and current life stress. J Trauma Dissociation. 2009;10(2):170-188. doi:10.1080/15299730802624585 19333847

[40] Nicol AL, Sieberg CB, Clauw DJ, Hassett AL, Moser SE, Brummett CM. The association between a history of lifetime traumatic events and pain severity, physical function, and affective distress in patients with chronic pain. J Pain. 2016;17(12):1334-1348. doi:10.1016/j.jpain.2016.09.003 27641311

[41] Kim-Mozeleski JE, Pandey R, Tsoh JY. Psychological distress and cigarette smoking among U.S. households by income: considering the role of food insecurity. Prev Med Rep. 2019;16:100983. doi:10.1016/j.pmedr.2019.100983 31516816 PMC6734047

[42] Skov-Ettrup LS, Nordestgaard BG, Petersen CB, Tolstrup JS. Does high tobacco consumption cause psychological distress? A mendelian randomization study. Nicotine Tob Res. 2017;19(1):32-38. doi:10.1093/ntr/ntw186 27613883

[43] Weinberger AH, Pacek LR, Sheffer CE, Budney AJ, Lee J, Goodwin RD. Serious psychological distress and daily cannabis use, 2008 to 2016: potential implications for mental health? Drug Alcohol Depend. 2019;197:134-140. doi:10.1016/j.drugalcdep.2019.01.010 30825793 PMC6440801

[44] Alpers SE, Pallesen S, Vold JH, Haug E, Lunde LH, Skogen JC, . The association between psychological distress and alcohol consumption and physical activity: a population-based cohort study. Front Psychiatry. 2023;14:1181046. doi:10.3389/fpsyt.2023.1181046 37426109 PMC10323831

[45] Levine JA, Gius BK, Boghdadi G, Connors GJ, Maisto SA, Schlauch RC. Reductions in drinking predict increased distress: between- and within-person associations between alcohol use and psychological distress during and following treatment. Alcohol Clin Exp Res. 2020;44(11):2326-2335. doi:10.1111/acer.14462 32945567 PMC7680417

[46] Al-Ghamdi S, Shubair MM, Angawi K, . Combined’ neck/back pain and psychological distress/morbidity among the Saudi population: a cross-sectional study. Front Psychol. 2022;13:870600. doi:10.3389/fpsyg.2022.870600 35519627 PMC9066093

[47] Kristoffersen ES, Aaseth K, Grande RB, Lundqvist C, Russell MB. Psychological distress, neuroticism and disability associated with secondary chronic headache in the general population—the Akershus study of chronic headache. J Headache Pain. 2018;19(1):1-12. doi:10.1186/s10194-018-0894-7 30116914 PMC6095768

[48] Nicholson RA, Houle TT, Rhudy JL, Norton PJ. Psychological risk factors in headache. Headache. 2007;47(3):413-426. doi:10.1111/j.1526-4610.2006.00716.x 17371358 PMC2408884

[49] McLachlan KJJ, Gale CR. The effects of psychological distress and its interaction with socioeconomic position on risk of developing four chronic diseases. J Psychosom Res. 2018;109:79-85. doi:10.1016/j.jpsychores.2018.04.004 29680578 PMC5959313

[50] Khan A, Pooja V, Chaudhury S, Bhatt V, Saldanha D. Assessment of depression, anxiety, stress, and quality of life in rheumatoid arthritis patients and comparison with healthy individuals. Ind Psychiatry J. 2021;30(suppl 1):S195-S200. doi:10.4103/0972-6748.328861 34908689 PMC8611570

