# Error in Article Information

**DOI:** 10.1001/jamanetworkopen.2024.60048

**Published:** 2025-01-14

In the Original Investigation titled “Psychological Distress Among Ethnically Diverse Participants From Eastern and Southern Africa,”^1^ published October 9, 2024, there was an error in the Article Information. Segun Fatumo was missing an affiliation with Precision Healthcare University Research Institute, Queen Mary University of London, United Kingdom. This article has been corrected.^1^
