# Supplementary material for: Psychological Distress Among Ethnically Diverse Participants From Eastern and Southern Africa
Source: JAMA Netw Open. 2024 Oct 9;7(10):e2438304. doi: 10.1001/jamanetworkopen.2024.38304 (PMC11581619; doi:10.1001/jamanetworkopen.2024.38304)
Supplement: Supplement 1. — eFigure. Distribution of Kessler Psychological Distress Scale Scores in the Study Participants eTable 1. Characterization of Study Population by Country eTable 2. Prevalence of Psychological Distress by Country eTable 3. Factors That Were Statistically Significant From the Proportional Odds Logistic Regression Analysis [file jamanetwopen-e2438304-s001.pdf]

Supplemental Online Content

Tindi KBB, Kalungi A, Kinyada E, et al. Psychological distress among ethnically diverse participants from Eastern and Southern Africa. *JAMA Netw Open*. 2024;7(10):e2438304. doi:10.1001/jamanetworkopen.2024.38304

eFigure. Distribution of Kessler Psychological Distress Scale Scores in the Study Participants
eTable 1. Characterisation of Study Population by Country
eTable 2. Prevalence of Psychological Distress by Country
eTable 3. Factors That Were Statistically Significant From the Proportional Odds Logistic Regression Analysis

This supplemental material has been provided by the authors to give readers additional information about their work.

© 2024 Tindi KBB et al. *JAMA Network Open.*

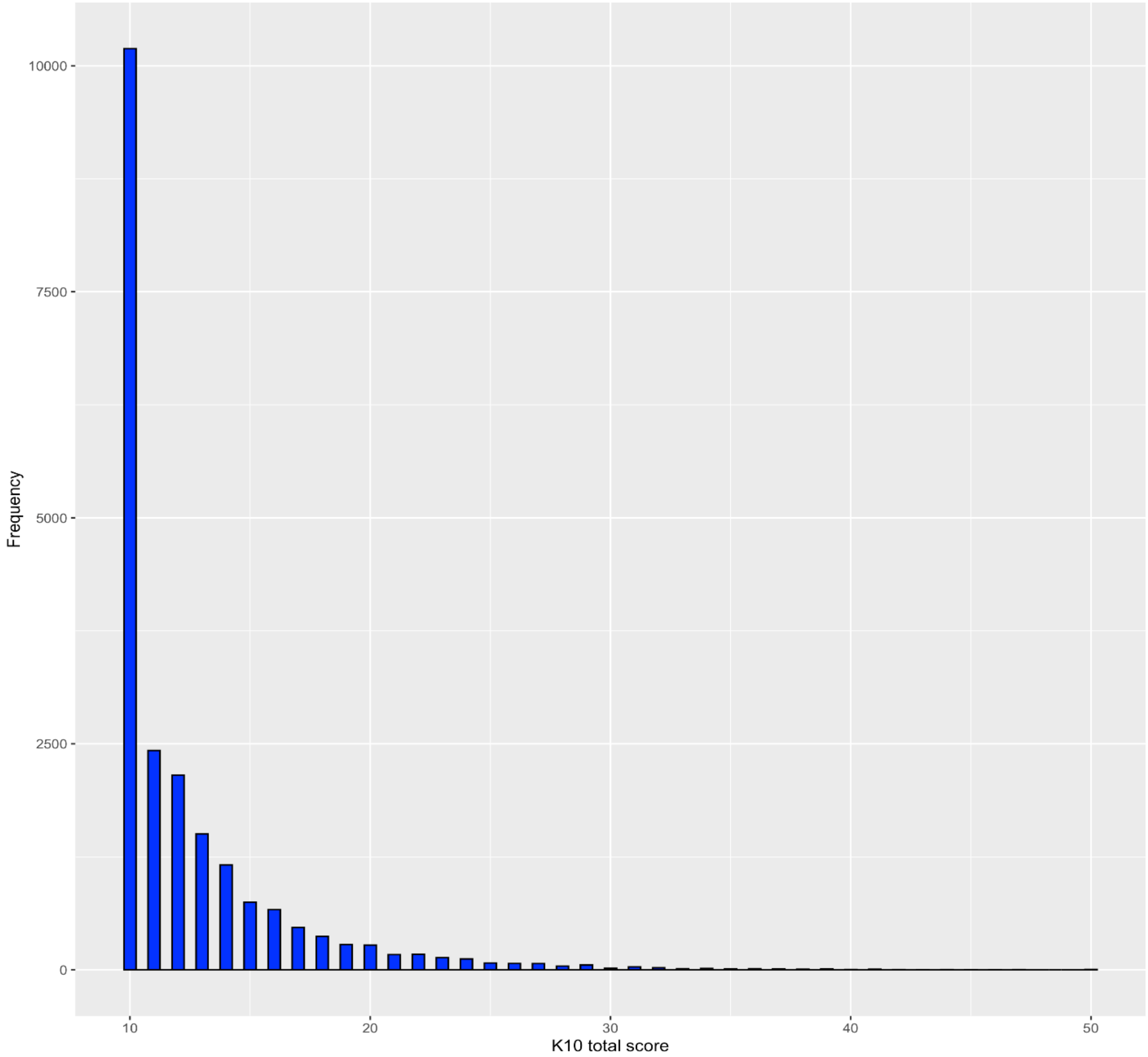

eFigure. Distribution of Kessler Psychological Distress Scale Scores in the Study Participants

© 2024 Tindi KBB et al. *JAMA Network Open.*

eTable 1. Characterisation of Study Population by Country

Population	Sample size	Sex		Age[a]			
		M	F	18-24	25-34	35-44	45+
Uganda	5991	2791	3200	1208	1806	1546	1278
Kenya	3932	2044	1888	761	1272	996	879
Ethiopia	6497	4202	2295	1002	2121	2033	1286
South Africa	4888	3059	1829	607	1439	1357	1414
**Total**	21308	12096	9212	3578	6638	5932	4857

Abbreviation: M, male; F, female

[a] Due to missing data for age per country; Uganda (n = 153), Kenya (n = 24), Ethiopia (n = 55) and South Africa (n = 71), these factors do not add up to the total sample size

© 2024 Tindi KBB et al. *JAMA Network Open.*

eTable 2. Prevalence of Psychological Distress by Country

Country	Mild PD (%)	Moderate PD (%)	Severe PD (%)
**Uganda**	2.9	0.7	0.6
**Kenya**	3.4	1.2	0.7
**Ethiopia**	1.8	0.5	0.3
**South Africa**	9.1	3.9	1.8

Abbreviation: PD, psychological distress

eTable 3. Factors That Were Statistically Significant From the Proportional Odds Logistic Regression Analysis

Risk factor	OR [a] (95% confidence interval)
**Sex**	
Female	1.63 (1.40-1.90)
**Traumatic life events**	
**Witnessed by participant**	
At least 1 event	1.70 (1.40-2.06)
**Experienced by participant**	
At least 1 event	2.32 (1.94-2.77)
**Alcohol use**	
Daily or almost daily	1.71 (1.24-2.36)
**Tobacco use**	
Daily or almost daily	1.41 (1.16-1.73)
**Cannabis use**	
Once or twice in the past 3 months	1.91 (1.31-2.80)
Daily or almost daily	1.81 (1.36-2.43)
**Arthritis**	
Has arthritis	1.55 (1.28-1.88)
**Chronic back or neck pain**	
Has pain	1.65 (1.44-1.92)
**Frequent or severe headaches**	
Has headaches	2.64 (2.33-3.01)
**Psychological distress progression**	
Control|mild	38.70 (22.46-66.67)
Mild|mod	124.50 (71.82-215.81)
Mod|severe	369.72 (210.14-650.51)

Abbreviation: OR, odds ratio

[a] Statistically significant associations from the analyses after correcting for multiple testing

© 2024 Tindi KBB et al. *JAMA Network Open.*
