# Supplementary material for: Psychological Distress Among Ethnically Diverse Participants From Eastern and Southern Africa
Source: JAMA Netw Open. 2024 Oct 9;7(10):e2438304. doi: 10.1001/jamanetworkopen.2024.38304 (PMC11581619; doi:10.1001/jamanetworkopen.2024.38304)
Supplement: Supplement 2. — Data Sharing Statement [file jamanetwopen-e2438304-s002.pdf]

Data Sharing Statement

Tindi. Psychological Distress Among Ethnically Diverse Participants From Eastern and Southern Africa. *JAMA Netw Open*. Published October 09, 2024.
doi:10.1001/jamanetworkopen.2024.38304

Data
Data available: No

Additional Information
Explanation for why data not available: This study made use of already collected data as part of the Neuropsychiatric Genetics in African Populations-Psychosis (NeuroGAP-Psychosis) study. The NeuroGAP-Psychosis study team will deposit and make all data available through the National Institute of Mental Health Data Archive at this site: https://nda.nih.gov/edit_collection.html?id=3805. This current study has no primary data to release independently and the parent study will release the data in their planned time.
